# The anticancer agent YC-1 suppresses progestin-stimulated VEGF in breast cancer cells and arrests breast tumor development

**DOI:** 10.3892/ijo.2012.1675

**Published:** 2012-10-24

**Authors:** CANDACE E. CARROLL, YAYUN LIANG, INDIRA BENAKANAKERE, CYNTHIA BESCH-WILLIFORD, SALMAN M. HYDER

**Affiliations:** 1 Department of Biomedical Sciences and Dalton Cardiovascular Research Center, University of Missouri, Columbia, MO 65211;; 2 IDEXX RADIL, Columbia, MO 65201, USA

**Keywords:** YC-1, progestins, progesterone receptors, VEGF, breast cancer

## Abstract

Recent epidemiological studies show that postmenopausal women taking estrogen-progestin hormone replacement therapy (HRT) have a higher risk of breast cancer than women on an HRT regimen lacking progestins. This may be related to the observation that progestin-treated breast cancer cells express and secrete high levels of vascular endothelial growth factor (VEGF), a potent angiogenic factor that promotes breast tumor growth. Anti-progestins such as RU-486 block this effect, indicating that progesterone receptors (PR) are involved in promoting VEGF induction; however antiprogestins cross-react with other steroid receptors which limits their clinical use. Alternative strategies are, therefore, needed to arrest the growth of progestin-dependent tumors. 3-(5′-hydroxymethyl-2′-furyl)-1-benzylindazole (YC-1), a novel anticancer drug initially developed as an inhibitor of HIF-1α, is currently undergoing preclinical trials against various forms of cancer. Since HIF-1α has recently been implicated in PR-mediated VEGF synthesis, we undertook studies to determine whether YC-1 inhibits progestin-dependent VEGF induction and tumor progression. Surprisingly, we found that YC-1 downregulated PR in human breast cancer cells, both *in vivo* and *in vitro*, thereby blocking progestin-dependent induction of VEGF and tumor growth. YC-1 also inhibited progestin-accelerated DMBA-induced mammary tumors in rats, properties which would likely render it effective against progestin-dependent tumors which frequently develop in post-menopausal women. We, therefore, propose that based on our observations, YC-1 warrants further investigation as a novel agent which could prove extremely useful as an anti-angiogenic chemotherapeutic drug.

## Introduction

Hormone replacement therapy (HRT) containing either estrogen alone or a combination of estrogen and a progestin such as medroxyprogesterone acetate (MPA), is a commonly-used treatment for menopause in women (1). Clinical studies show that estrogen-based HRT is associated with increased risk of uterine cancer, while there is an elevated risk of breast cancer, metastasis and mortality in women undergoing estrogen/progestin-based HRT (2–4). Our recent studies show that progestins induce *in vitro* expression of vascular endothelial growth factor (VEGF), a potent angiogenic growth factor, in a subset of human breast cancer cells that express mutant p53 (5,6) and stimulate breast tumor growth *in vivo*(7,8). Conversely, the anti-progestin RU-486 and anti-VEGF antibodies inhibit secretion and function of VEGF *in vitro* and block breast tumor growth *in vivo*(7,8). These results support the hypothesis that pharmacological use of progestins increases the risk of progesterone receptor (PR)-dependent breast cancer in post-menopausal women by a mechanism involving induction of VEGF. Though anti-progestins such as mifepristone or RU-486 suppress progestin-dependent actions, their use is limited due to a number of side effects that occur as a result of cross-reactivity with other steroid receptors (9). Thus other means of controlling PR mediated effects are being explored.

Hypoxia-inducible factor-1α (HIF-1α), is a basic helix-loop-helix transcription factor that regulates expression of VEGF and other genes that modulate growth, survival and metastasis of tumor cells under conditions of hypoxia (10,11). Under normoxic conditions, HIF-1α is rapidly degraded, but under hypoxic conditions, HIF-1α dimerizes with and is stabilized by HIF-1β, and the HIF-1α, β heterodimer actively regulates expression of promoters containing its cognate response element, hypoxia-response element (HRE) (12) that is also located in the VEGF promoter. Several studies suggest that HIF-1α regulates expression of VEGF in cancer cells (13,14). 3-(5′-hydroxymethyl-2′-furyl)-1-benzylindazole (YC-1) (15) was initially developed as a specific HIF-1α inhibitor which interferes with binding of HIF-1α to promoter regions of its target genes (10). However, YC-1 has been found to possess additional properties; it is also a nitric oxide synthetase (NOS)-independent activator of soluble guanylyl cyclase (sGC), which has been used clinically to treat thrombosis and hypertension (16), and it blocks NF-κB activity in tumor cells (17). Importantly, previous studies suggest that YC-1 may have significant anti-tumor activity by targeting HIF-1α and NF-κB transcription factors (18).

Recent studies in a bovine model have shown that HIF-1α plays a role in PR mediated VEGF induction (13). With this in mind we used cell culture techniques and rodent models to examine, both *in vitro* and *in vivo*, the capacity of YC-1 as a HIF-1α inhibitor which might be used to control progestin-stimulated PR-dependent VEGF secretion and progression of progestin-dependent breast cancer. Breast cancer cells were exposed to YC-1 *in vitro* or *in vivo* and their growth and tumorigenic properties examined. Unexpectedly we made the novel observation that, both *in vivo* and *in vitro*, YC-1 downregulates PR in human breast cancer cells. Furthermore, YC-1 arrests the progression of progestin-dependent breast cancer cells *in vivo* by preventing VEGF induction which depends upon progestin activity.

## Materials and methods

### Materials

Human T47-D and BT-474 breast cancer cell lines were obtained from ATCC (Manassas, VA). 3-(5′-hydroxymethyl-2′-furyl)-1-benzylindazole) (YC-1), was purchased from Biomol International, LP (Plymouth Meeting, PA). Phenol red-free DMEM/F12 medium, phosphate-buffered saline and 0.05% trypsin-EDTA were purchased from Invitrogen Corporation and Life Technologies (Grand Island, NY). Fetal bovine serum (FBS) was obtained from JRH Biosciences (Lenexa, KS).

### Cell viability assay

T47-D and BT-474 human breast cancer cells were exposed to YC-1 (1–100 *μ*M) for 18 h and cell viability was tested using the sulforhodamine B (SRB) assay (19,20).

### Cell culture and VEGF ELISA

Cells were grown in DMEM/ F12 + 10% FBS for routine culture. Media was replaced with DMEM/F12 containing 5% dextran-coated charcoal (DCC)-treated serum for 24 h prior to treatment of cells in triplicate with progestins and inhibitors for a further 18 h. Media was collected and ELISA for VEGF performed using a VEGF ELISA kit from R&D Systems, Inc. (Minneapolis, MN) as previously described (21–23). According to the manufacturer’s protocol, the minimum detectable concentration of VEGF is less than 5 pg/ml, the intra-assay precision has a coefficient of variance (CV %) between 3.5–6.5%, while the CV % of the inter-assay ranges from 5.0 to 8.5%.

### Bicinchoninic acid (BCA) protein assay

BCA assay using bovine serum albumin as a standard was used to determine total protein concentrations as described before (21–23). All samples were analyzed in duplicate.

### Preparation of whole cell extract for western blot analysis

Whole cell extracts were prepared with a nuclear extract kit (Active Motif). Briefly, cells were grown in 100 mm culture dish overnight and then treated with DMEM/F12 containing 5% FBS-DCC serum for 24 h. Cells were then treated with 10 nM MPA alone and in the presence of 100 *μ*M YC-1, or YC-1 alone for 6 h. At the end of treatments, cells were washed with ice-cold PBS containing phosphatase inhibitors (with kit), harvested by gentle scraping with a cell lifter, and centrifuged for 5 min at 200 × g at 4°C. Cell pellets were re-suspended in complete lysis buffer (provided in the TransAm kit; 1 mM DTT and 1% protease inhibitor cocktail was added prior to use) and incubated on ice for 30 min with shaking. Samples were centrifuged at 14,000 × g for 15 min, and the supernatant was transferred to a microcentrifuge tube, aliquoted, and stored at −80°C.

### Western blotting

Proteins (50 *μ*g per lane) were separated in a 10% NuPAGE Bis-Tris Gel (Invitrogen, Carlsbad, CA). Electrophoresis was performed at 120 V for 1.5 h using NuPAGE MOPS-SDS Running Buffer. Separated proteins were transferred to polyvinylidene difluoride membranes (Bio-Rad Laboratories, Hercules, CA) at 25 V for 25 min (Dry transfer). The blots were blocked for 1 h at room temperature (RT) in 5% non-fat dry milk in TBS containing 0.1% Tween-20 (TBS-T), and incubated with anti-PR (1:200) and anti-HIF-1α (1:150) antibodies overnight at 4°C. The blots were washed 3 times with TBS-T and incubated with secondary antibody for 1 h at RT. The blots were then washed 7 times (8 min each) with TBS-T and immunoreactive bands were visualized using an ECL plus detection kit (Amersham, Pharmacia Biotech, Arlington Heights, IL). Membranes were stripped and re-blotted for β-actin (Sigma, St. Louis, MO), which was used as a control for protein loading.

### RNA extraction and RT-PCR

Cells were treated with progestin (10 nM) ± 100 *μ*M YC-1 for 6 h at 37°C. Ultraspec RNA reagent (1 ml) (Biotecx Products, Oxon, UK) was added to each plate and RNA was purified as described by the manufacturer. Pellets containing purified RNA were re-suspended in DEPC treated water. RNA was DNase treated prior to RT-PCR which was performed as described previously (23). VEGF and β-actin primer pairs were purchased from R&D Systems, Inc.; DNA sequences for these are propriety. Forward 5′-3′: ATGAGAAGTATGACAACAGCC, and reverse 5′-3′: TGAGTCCTTCCACGATACC were used for GAPDH. The following cycle was employed; 60°C for 30 min for cDNA synthesis, 94°C for 2 min for denaturing and 40 cycles of 94°C for 15 sec, 50–55°C for 30 sec and 68°C for 1 min for amplification. Final extension was for 7 min at 68°C.

### DMBA-induced mammary tumors

Intact virgin female 40–45 day old Sprague-Dawley rats (Harlan Sprague-Dawley, Indianapolis, IN) were housed according to the guidelines of the Association for Assessment and Accreditation of Laboratory Animal Care under conditions of 12-h light/dark cycles and *ad libitum* access to food and water. All surgical and experimental procedures were in accordance with the ‘Guide for Care and Use of Laboratory Animals’ (NIH publication 85-23). Animals were given a single dose of 20 mg of DMBA in peanut oil via gavage on day 0. On day 30, animals were anesthetized and MPA pellets were implanted subcutaneously on the dorsal surface (7). On day 68, YC-1 (3.75 mg/day) (18), or vehicle, was administered to animals via tail vein injection for 5 days. Animals were palpated and tumors measured 2–3 times weekly. Mammary tumor tissues were collected at the time of sacrifice for immunohistochemistry (IHC) analysis. In order to determine blood vessel perfusion within tumors, animals were injected with 0.5 mg Texas red-tomato lectin conjugate 10 min prior to sacrificing, as described (24).

### Xenograft tumor study

Five to six week old female athymic nu/nu mice, purchased from Harlan Sprague-Dawley, Inc., were housed in a laminar airflow cabinet under specific pathogen-free conditions. Nude mice were inoculated with 17-β-estradiol pellets (60-day timed release, 1.7 mg) 24–48 h before implantation of T47-D or BT474 cells in both flanks as described previously (8). In this model tumor cells initially grow but then spontaneously regress and the regression is rescued with progestins (8). In the experiments reported in this study, when tumors regressed to approximately 50% of their peak volume following tumor cell injection (6–10 days), MPA pellets (10 mg/60-day release) were implanted. Once tumor volume reached 80–120 mm^3^, animals received 10 daily treatments of YC-1 (600 *μ*g/mouse, i.p.) (18), or vehicle alone. Tumor volume and animal weights were measured every 3 days. At the end of the experiment, animals were sacrificed and tumors collected 2 h following final treatment with YC-1 for IHC analysis.

### Histology and immunohistochemisty

For both rat and mouse tumors, tissues were fixed overnight in 4% paraformaldehyde and processed for paraffin infiltration and embedding. Sections (5 *μ*m) were mounted on ProbeOn Plus microscope slides (Fischer Scientific, Inc., Pittsburgh, PA) and routinely stained with hematoxylin and eosin (H&E) or prepared for immunohistochemical labeling. Prior to immunohistochemistry, sections were dewaxed in xylene, rehydrated through graded concentrations of ethanol, rinsed (wash buffer, Dako Carpinteria, CA) prior to immersion and heated in 10 mmol/l citrate buffer (pH 6.0) for 20 min for heat-induced epitope retrieval. This tissue treatment was performed for PR, ERα, ERβ, CD34 and VEGF immuno-labeling. Slides were cooled for 20 min, treated with 3% H_2_O_2_ (to inactivate endogenous peroxidase activity) and rinsed prior to incubation with 5% bovine serum albumin for 20 min. Sections were then incubated for 60 min at room temperature with each of the following antibodies: anti-PR antibody [1:50 dilution of a rabbit anti-human PR polyclonal antibody (A0098), Dako], anti-ERα [1:300 dilution of a rabbit anti-ERα polyclonal antibody (sc-542), Santa Cruz Biotechnology, Inc., Santa Cruz, CA], anti-ERβ [1:50 dilution of a mouse anti-ERβ monoclonal antibody (MCA1974s) AbD Serotec, Raleigh, NC], anti-CD34 (1:100 dilution of a goat anti-CD34 polyclonal antibody), and an anti-VEGF antibody [1:100 dilution of a rabbit anti-VEGF polyclonal antibody (sc-152); Santa Cruz Biotechnology, Inc.]. Sections were then washed, incubated for 30 min with a biotinylated secondary antibody [rabbit anti-mouse IgG (Dako) for anti-ERβ labeled sections and a rabbit anti-goat IgG (Dako) for the anti-CD34 probed sections] and then for 30 min with a streptavidin-linked horseradish peroxidase product (Dako). Sections for PR, ERα and VEGF were incubated with EnVision, a horseradish peroxidase-labeled polymer conjugated to anti-rabbit antibodies (Dako). Bound antibodies were visualized following incubation with 3,3′-diaminobenzidine solution (0.05% with 0.015% H_2_O_2_ in PBS; Dako) for 3–5 min. Sections were counterstained with Meyer’s hematoxylin, dehydrated, and coverslipped for microscopic examination.

### Texas red conjugated-tomato lectin

Sections (8 *μ*m) of frozen tumors were made using a cryostat. After rinsing, sections were incubated with 4% paraformaldehyde, rinsed again, and mounted with DAPI (Vectashield Hardset with DAPI, Vector Lab, Burlingame, CA) and coverslipped. In order to visualize Texas red-labeled tomato lectin staining, samples were imaged with a 590 nm bandpass filter at 1/2 second exposure.

### Statistical analysis

For VEGF analysis, data were analyzed by one-way ANOVA. Data were checked for normality and homogeneity of variance. Since a number of analyses did not satisfy these assumptions, data were ranked and ANOVA performed as outlined by Conover and Iman (25). Fisher’s protected least significant difference (LSD) was performed to determine treatment differences, as suggested by Chew (26). All significance was based on the ranked transformations; however treatment values were presented as actual values. For DMBA studies, a difference in the growth curve of tumor size was compared via t-test. For nude mice studies, points along the tumor volume curve were compared via one-way ANOVA. FoveaoPro 3.0^®^ software analysis was used to determine positive staining by area in immunohistochemical studies. One-way ANOVA was used to statistically compare VEGF, CD34, and PR staining differences among experimental groups. For all statistical comparisons, p<0.05 was regarded as statistically significant.

## Results

### YC-1 reduces cell viability of human breast cancer cells in a concentration dependent manner

**Figure 1.**
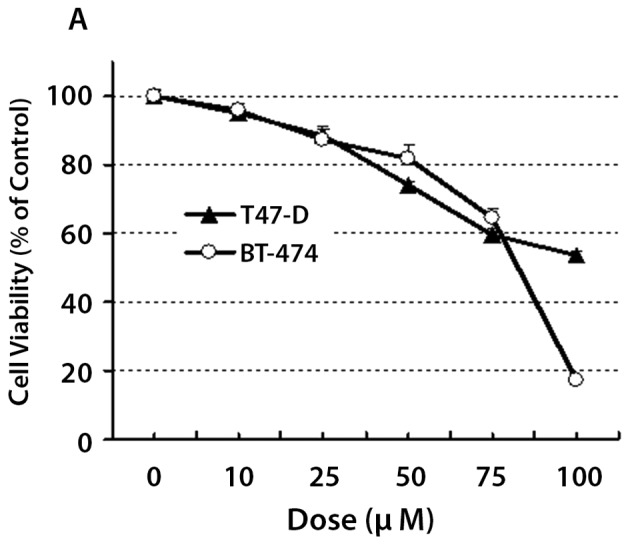

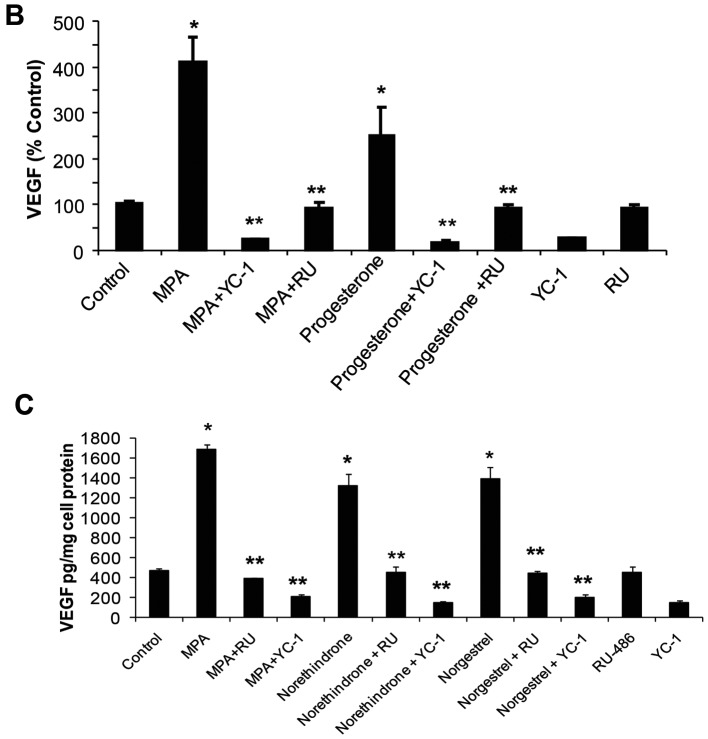
YC-1 inhibits cell viability and progestin-induced VEGF secretion in human breast cancer cells (n=6 for each concentration). (A), T47-D and BT-474 cells were treated with indicated concentrations of YC-1 for 18 h and cell viability was determined using the SRB assay. ^*^Significantly different from control (p<0.001, ANOVA). (B), T47-D cells were pre-treated for 30 min with 1 *μ*M RU-486, or 100 *μ*M YC-1 and then treated with 10 nM progesterone or MPA or with synthetic progestins (C). Media was collected after 16–18 h and analyzed for VEGF using ELISA as described in Materials and methods (n=3–12). One-way ANOVA was used to statistically analyze data. ^*^Significant induction compared with control. ^**^Significant inhibition compared with appropriate progestin control, p<0.05. The control value for VEGF in Fig. 2A represents 496±51 pg/mg cellular protein.

T47-D and BT-474 cells were treated with increasing doses of YC-1 for 18 and cell viability monitored using the SRB assay (Fig. 1A). YC-1 effectively reduced cell viability in a concentration-dependent manner in a similar fashion to its effect on prostate cancer cells (17). In keeping with a previous study (17), we used a dose of 100 *μ*M YC-1 for subsequent *in vitro* analysis that involved 6–18 h exposure to the drug. Since YC-1 was extremely potent against BT-474 cells at this concentration, we performed subsequent *in vitro* studies using T47-D cells in an effort to study the molecular mechanisms underlying the effects of YC-1.

### YC-1 inhibits progestin-stimulated secretion of VEGF from T47-D cells

To determine whether YC-1 plays a role in progestin-stimulated secretion of VEGF from human breast cancer cells, T47-D cells were incubated for 30 min with 10- or 100 *μ*M YC-1 or 1 *μ*M anti-progestin RU-486 [anti-progestin (21–23)]. Following this initial incubation, 10 nM MPA (most commonly used synthetic progestin in HRT) or 10 nM progesterone was added for 18 h and VEGF secreted into the culture medium was quantified by ELISA. As reported previously by us (21–23), expression of VEGF was 3- to 4-fold higher in MPA and progesterone-treated T47-D cells compared with controls; VEGF induction was completely inhibited by a 100-fold excess of RU-486, indicating the involvement of PR (Fig. 1B). Interestingly, while 10 *μ*M was without effect, 100 *μ*M YC-1 completely inhibited the induction of VEGF by progestin in T47-D cells in the time frame tested (Fig. 1B and data not shown). YC-1 also reduced basal levels of VEGF secretion from T47-D breast cancer cells, most likely by inhibiting HIF-1α, which is the major transcription factor required for VEGF induction, though this remains to be proven. Importantly, YC-1 also blocked the effect on VEGF secretion of other synthetic progestins used in HRT, including norethindrone and norgestrel (Fig. 1C).

### YC-1 inhibits progression of MPA-dependent human breast cancer xenografts in nude mice

**Figure 2.**
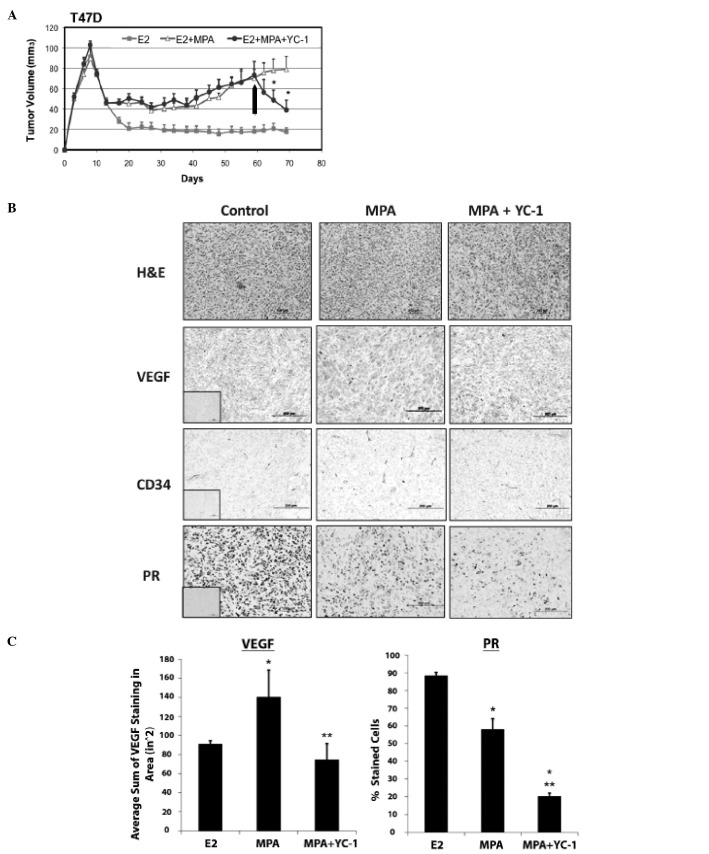
YC-1 inhibits progression of MPA-dependent breast cancer xenograft tumors in nude mice. (A), Xenograft tumors derived from T47D-cells were grown as described in Materials and methods. Tumor bearing mice were treated with YC-1 or vehicle alone (i.p., arrow) as described in Materials and methods (n=5, 10 tumors per group). ^*^Significantly different from controls as analyzed by one-way ANOVA, p<0.05. (B), Immunohistochemical analysis of MPA-dependent, T47D-derived tumors. H&E, VEGF, CD34 and PR expression were analyzed in control (mice containing E_2_ pellets), untreated (E2 + MPA + vehicle) and treated (E_2_ + MPA + YC-1) tumors. Collected tumor tissues were sectioned and immunostained for the proteins shown, as described in Materials and methods. Insets represent negative controls with no primary antibody staining for each antibody. Original magnification, ×20. (C), Quantification of VEGF and PR immunostaining from tissues analyzed in (B). Four fields from each tumor section were analyzed to control for variation due to cellularity (control, n=3 tumors; MPA + vehicle, n=3 tumors; MPA + YC-1, n=4 tumors). VEGF staining (left panel): positive VEGF staining was quantified as the number of VEGF-positive pixels in different fields using FoveaoPro 3.0 analysis software. Error bars represent SEM. ^*^Statistical significance compared to control (p<0.001; ANOVA). ^**^Statistical significance compared to MPA + vehicle (p<0.001; ANOVA). PR staining (right panel) percentage of positive cells in 6 fields of each section was counted. Error bars represent SEM (^*^p<0.001).

We previously developed a mouse xenograft model for identifying and characterizing factors that promote or prevent progestin-accelerated breast cancer (8). In the present study we used this model to examine the effects of YC-1 on growth of MPA-stimulated T47-D xenograft tumors. In this experimental design (Fig. 2A), xenograft tumors were allowed to grow to a volume of 80–120 mm^3^, after which tumor-bearing mice were treated with YC-1 [600 *μ*g/mouse/day (18)] for 10 days. Tumor volume and animal weight were measured every third day throughout the experiment. Fig. 2A shows clearly that YC-1 inhibited growth and caused regression of T47-D xenograft tumors. At the chosen dose level, YC-1 was well tolerated and no signs of toxicity were detected in YC-1-treated mice (data not shown). Similar experiments conducted using nude mice bearing xenograft tumors derived from tamoxifen-resistant Her-2-neu enriched BT-474 breast cancer cells (27) showed that YC-1 strongly and rapidly inhibited growth of BT-474 tumor xenografts that were exposed to the natural hormone progesterone (data not shown).

Immunohistochemical studies were performed on MPA-exposed T47-D xenograft tumors from YC-1 and vehicle-treated mice. Tissues for these studies were isolated from animals sacrificed 2 h after the final injection with YC-1. As expected, VEGF staining was higher in tumors from MPA-treated mice, and exposure to YC-1 reduced VEGF expression by a statistically significant amount (Fig. 2B and C). Although the difference was not statistically significant, YC-1 also appeared to reduce expression of CD34 in tumors from MPA-treated mice (Fig. 2C; 16±2.3, n=3, MPA + YC-1 vs. 20±5.5, n=5, MPA); this finding is consistent with the possibility that YC-1 inhibits angiogenesis in T47-D xenograft tumors under the chosen experimental conditions.

### YC-1 reduces PR levels in vivo

Immunohistochemical analysis of PR expression was also carried out on T47-D xenograft tumors from YC-1 treated mice. Fig. 2B and C (right panel) shows that PR levels were significantly lower in tumors from mice treated with MPA or MPA/YC-1 than in tumors from control animals treated with only E2 [all animals were administered E2 by implant to facilitate initial tumor formation; however E2 alone does not lead to tumor growth under the conditions used for growing P-dependent tumors (8,27)]. The difference in PR expression between each of these three groups was statistically significant, suggesting that different mechanisms are responsible for repression of PR by YC-1 and MPA. Additional studies showed that the expression of ERα and ERβ is not modulated by YC-1 or MPA in the context of this mouse xenograft tumor model (data not shown).

### YC-1 reduces PR levels in vitro and inhibits progestin-dependent transcription of the VEGF gene

**Figure 3.**
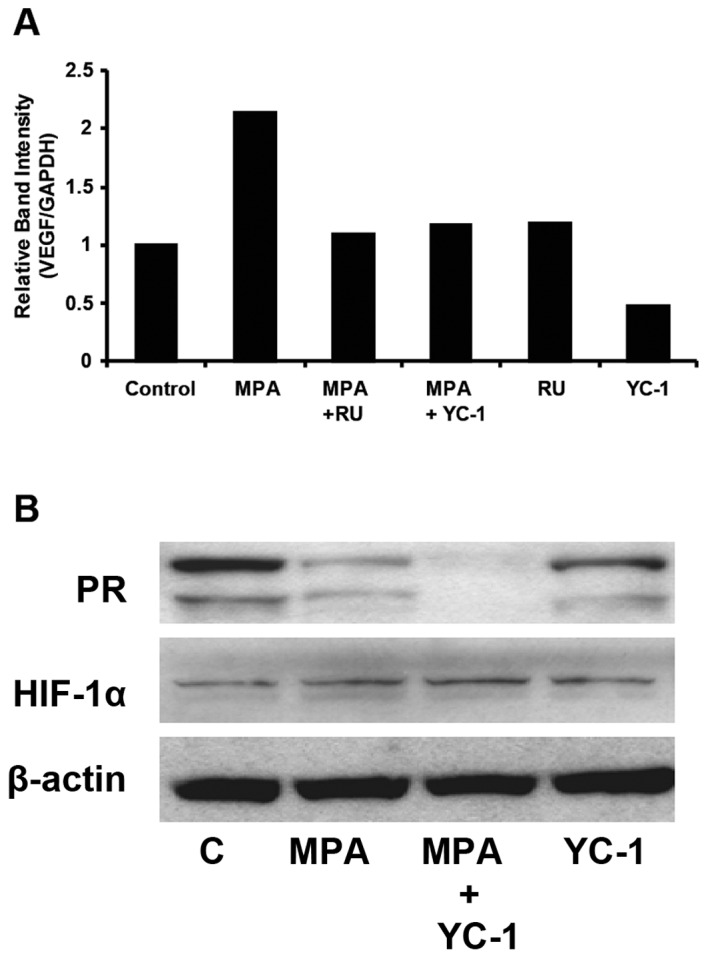
(A), YC-1 inhibits progestin-induced VEGF transcription in T47-D human breast cancer cells. Cells were treated with 100 *μ*M YC-1 for 6 h and RT-PCR was used to detect VEGF mRNA using 1 *μ*g of RNA from treated cells, as described in Materials and methods. Results shown are mean of two experiments. (B), Western blot analysis of T47-D cell nuclear extract. T47-D cells were treated with 10 nM MPA alone or in the presence of 1 *μ*M RU-486, 100 *μ*M YC-1 or 100 *μ*M 2ME2, or with inhibitors alone for 6 h. Nuclear extracts were collected as described in Materials and methods and 20 *μ*g of protein was loaded and probed for HIF-1α, progesterone receptor A and B and β-actin. In the progesterone receptor (PR) lanes, upper band represents PRB while the lower band represents PRA. RU = RU-486.

In order to confirm *in vivo* observations that YC-1 downregulates VEGF and PR, we conducted *in vitro* assays and examined the effect of YC-1 on transcription of VEGF mRNA in MPA-treated cells. For this study, T47-D cells were incubated for 30 min in the presence of 100 *μ*M YC-1 or 1 *μ*M RU-486, followed by 6 h with 10 nM MPA (23). YC-1 and RU-486 completely blocked progestin-stimulated VEGF transcription (Fig. 3A). The effect of YC-1 on levels of PR and HIF-1α was also determined in treated and untreated T47-D cells. Fig. 3B demonstrates that exposure to YC-1 for 6 h ± MPA resulted in reduced expression and/or stability of PR in T47-D cells. A similar effect was observed in cells exposed to progestins which activate PR [coupled to receptor degradation (30) and stimulates secretion of VEGF (21–23)]. PR expression was lower in those cells treated with a combination of MPA and YC-1 compared with cells treated with only YC-1, suggesting different mechanisms of action. Such an effect was also observed *in vivo* (see above). YC-1 did not promote degradation of HIF-1α in T47-D breast cancer cells under the conditions employed, indicating that PR loss did not occur as a result of a generalized cytotoxic effect of YC-1. β-actin was used as loading control.

### YC-1 inhibits progression of DMBA-induced MPA-driven mammary tumors and reduces expression of VEGF in tumor cells

**Figure 4.**
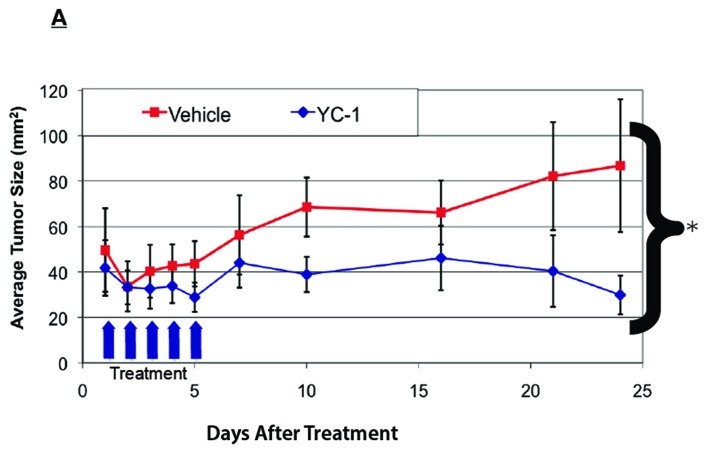

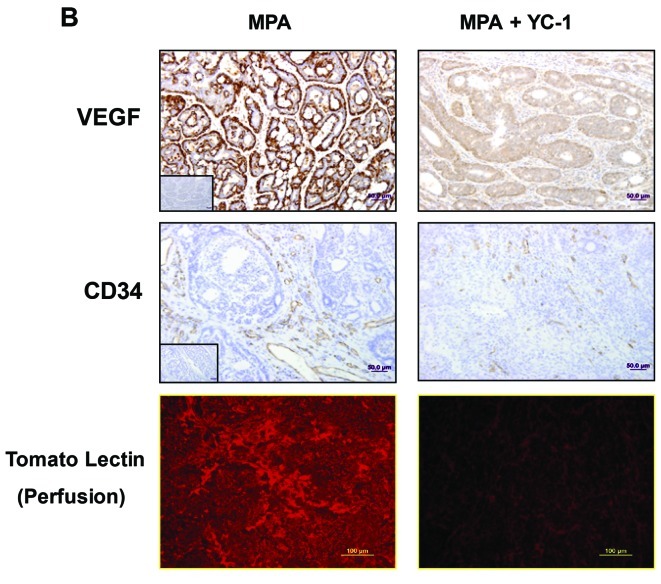
(A), YC-1 suppresses progestin-induced progression of DMBA-induced mammary tumors in rats. Sprague-Dawley rats were treated with 20 mg DMBA via gavage. After 4 weeks, 25 mg/60-day timed release pellets of MPA were implanted as described in Materials and methods. Beginning on day 68, 3.75 mg YC-1, or vehicle, was administered to animals for a further 5 days via tail vein injection. Error bars represent SEM, n=3 animals for control group (7 tumors) and 4 animals in the YC-1 treatment group (9 tumors). ^*^Significant difference between control and YC-1 treated group as analyzed by t-test, p<0.05. (B), Immunohistochemical analysis of tumor tissues from DMBA animal studies shown in (A). Collected tumor tissues were sectioned and immunostained for the designated protein as described in Materials and methods. Insets represent no antibody control. Original magnification, ×20. Bar represents 50 *μ*m except in figures containing Tomato lectin staining, where bar represents 100 *μ*m.

Our previous studies showed that MPA stimulates expression and secretion of VEGF and accelerates the development of DMBA-induced mammary tumors in female Sprague-Dawley rats (7). In the present study we sought to determine whether YC-1 inhibits mammary tumor progression in this model system. DMBA was administered to female Sprague-Dawley rats (45–50 days old), MPA pellets (25 mg, 60-day release) were implanted subcutaneously on day 28 and tumors were allowed to develop. On day 68, tumor-bearing animals were treated with YC-1 (3.75 mg/rat/day) as described in Materials and methods. YC-1 administration continued for 5 consecutive days; control animals were treated with vehicle (Fig. 4A). At the commencement of YC-1 treatment tumors ranged in size from 2 to 100 mm^3^ (tumors grow at different rates in this animal model system and new tumors develop at various times resulting in variations in size). Animals were weighed to monitor drug toxicity. Tumors were measured and palpated daily during the course of treatment and 2–3 times per week after the cessation of daily injections until the end of the study. As expected a continuous increase in tumor size occurred in vehicle-treated animals, while YC-1 inhibited growth and progression of tumors with an original size up to 100 mm^3^, the largest size of tumor treated (Fig. 3A). The effect of YC-1 lasted for 20 days after the last injection of the drug, at which point the experiment was terminated and tumors removed for immunohistochemical analysis. Based on animal weight, YC-1 was not toxic at this dose (data not shown).

Although the histology of DMBA-induced mammary tumors from animals administered YC-1 did not differ greatly from those given vehicle, immunohistochemical analysis showed a significant reduction in VEGF staining in tumors obtained from YC-1 treated animals (Fig. 4B, upper panel), together with a corresponding decrease in CD34 staining (Fig. 4B, middle panel), suggesting a reduced number of blood vessels. The reduction in CD34-positive blood vessels was not statistically significant; however, blood vessels in tumors collected from YC-1 treated rats were also smaller in diameter, further suggesting a reduced blood supply compared with tumors from control animals not administered YC-1. This was confirmed by a significantly reduced rate of blood perfusion in tumors from YC-1-treated rats (Fig. 4B, lower panel; see Materials and methods for experimental details). As noted above, YC-1 had no effect on the levels of expression of PR, ERα or ERβ in DMBA-induced mammary tumors in rats (data not shown). However, receptor expression was examined at just one time point, twenty days after the last injection of YC-1, which may allow PR to recover. Consequently additional studies of hormone receptor expression in YC-1 treated DMBA-initiated rats are needed.

## Discussion

Recently a number of studies and clinical trials have shown that HRT which contains a progestin component is associated with a higher risk of metastatic breast cancer than HRT lacking progestin (1–4). Anti-progestins, such as RU-486, cannot be used to mitigate this risk, because of their lack of specificity and severe adverse effects (9). Because a large number of postmenopausal women who are already at significant breast cancer risk are routinely exposed to progestin-containing HRT, there is an urgent need to develop viable alternative strategies that can be rapidly developed for clinical use, to lower the risk of or even prevent breast cancer. Here we report the results of preclinical studies in which we evaluated the potential of YC-1, a recently developed anticancer agent, to prevent the progestin-dependent progression of human breast cancer.

Progestins induce VEGF in breast cancer cells (29) in a PR-dependent manner and cause tumor progression and metastasis in animal models (8,27). VEGF expression within tumor cells is under the control of a number of essential transcription factors, one of the most important being HIF-1α, which regulates survival genes, including VEGF (14,15). Higher levels of HIF1α and VEGF are associated with increased risk of tumor metastasis (27,30). Interestingly, recent studies also provide evidence that HIF-1α is involved in hormone mediated regulation of VEGF (13,31). Thus HIF-1α appears to play a central role in the regulation of VEGF by steroid hormones in reproductive tissues, though a direct role of HIF-1α in nuclear receptor mediated events remains to be established. Since the induction of VEGF by progestins is well established in breast cancer cells (8,9,21–23,29) and it is possible that one mechanism through which this occurs involves HIF-1α, we undertook studies using both naturally-occurring and synthetic progestins and found that YC-1 inhibits induction of VEGF in both T47-D and BT-474 cells. Synthetic progestins which are used in various formulations worldwide as HRT all induce VEGF and have the potential to promote tumor metastasis (27). Although previous studies have shown that YC-1 inhibits angiogenesis (32), ours is the first to demonstrate that it blocks progestin-dependent angiogenesis. We propose therefore that YC-1 could be a useful drug which might be used clinically to prevent progestin-dependent tumor growth and metastasis. To lend further support to the notion that YC-1 might prove effective against progestin-dependent breast disease, we found that it also suppressed PR levels in breast cancer cells, both *in vitro* and *in vivo*. The effect of YC-1 could therefore be 2-fold; inhibition of HIF-1α and downregulation of PR, making it a uniquely suitable small molecule drug for combating those forms of breast cancer which are largely dependent on the actions of progestins to stimulate the production of VEGF. Further studies are required to determine the molecular mechanism through which YC-1 brings about the loss of PR, since it is possible that HIF-1α regulates PR and YC-1 shuts down PR synthesis due to loss of HIF-1α activity.

This study demonstrates that YC-1 effectively inhibits the growth of progestin-accelerated xenograft tumors derived from T47-D or BT-474 cells in mice and DMBA-induced tumors in rats, supporting the notion that it possesses significant potential as an anti-breast cancer agent *in vivo*. Although the exact mechanism by which YC-1 exerts its *in vivo* effects has not yet been established, we propose that YC-1, by inhibiting progestin-mediated VEGF-dependent angiogenesis, reduces blood flow to xenograft tumors in these experimental model systems. Alternatively, YC-1-mediated downregulation of PR could play a key role in the anti-tumor effectiveness of this HIF-1α inhibitor, a possibility which remains to be tested. Since ER levels were not affected by YC-1, it is likely that combination therapy using YC-1 and an anti-estrogen might be more effective than therapy using either compound alone, another scenario that requires further study.

In the Sprague-Dawley rat DMBA-induced progestin-accelerated tumor model, YC-1 prevented the progression of progestin-dependent tumors and inhibited expression of VEGF, but did not downregulate PR. However, since these tumors were analyzed several days after the end of YC-1 treatment it is possible that PR was re-expressed in tissues over time. While there was no significant reduction in the number of blood vessels in tumors from YC-1-treated animals, blood vessels were smaller and had reduced capacity for perfusion (Fig. 4B), a phenomenon we also observed in other experimental models (24,33). It therefore appears that YC-1 inhibits tumor growth in the DMBA-induced progestin-accelerated tumor primarily by downregulating VEGF. However, additional analysis of the kinetics of PR expression during studies such as these is warranted.

In conclusion, this study provides strong evidence that YC-1, a potent HIF-1α inhibitor, may be a useful pharmacologic agent which might be used to treat and possibly prevent PR-dependent breast cancer as a result of promoting PR loss. This could be especially valuable in a clinical context to mitigate the increased breast cancer risk associated with progestin-containing HRT.
